# Multi-layered functional genomics prioritizes candidate effectors and regulatory mechanisms of ankylosing spondylitis

**DOI:** 10.3389/fimmu.2026.1859310

**Published:** 2026-06-01

**Authors:** Fanyu Meng, Ling Chen, Meng Li, Qingqing Zhang, Peiying Wang, Ruifeng Lin, Jiani Liu, Zhao Yuan, Kexin Chen, Zhaoxia Li, Yetong Xie, Abuduwahapu Aierken, Furkat Yalkun, Chulan Li, Yuan Ma, Jianhui Chen, Ziwen Xu, Fei Zhong

**Affiliations:** 1 The Sixth School of Clinical Medicine, Xinjiang Medical University, Urumqi, Xinjiang, China; 2 Xinjiang Institute of Spinal Surgery, The Sixth Affiliated Hospital of Xinjiang Medical University, Urumqi, Xinjiang, China; 3 Department of Rheumatology and Immunology, Nanfang Hospital, Southern Medical University, Guangzhou, China; 4 Department of Traditional Chinese Internal Medicine, School of Traditional Chinese Medicine, Southern Medical University, Guangzhou, China; 5 The Second School of Clinical Medicine, Southern Medical University, Guangzhou, China; 6 The Second Affiliated Hospital of Guilin Medical University, Guilin, China; 7 The School of Public Health, Guangzhou Medical University, Guangzhou, China; 8 Department of Neurology, The First Affiliated Hospital of Bengbu Medical University, Bengbu, China; 9 The First School of Clinical Medicine, Southern Medical University, Guangzhou, China; 10 Department of Nephrology and Rheumatology, The Affiliated Guangzhou Hospital of Traditional Chinese Medicine, Guangzhou University of Chinese Medicine, Guangzhou, China

**Keywords:** AlphaGenome, ankylosing spondylitis, causal transcriptome-wide association, Geneformer, siRNA knockdown, variant effect prediction

## Abstract

**Background:**

Ankylosing spondylitis (AS) is a chronic inflammatory arthropathy with heritability estimated at approximately 90%, yet the effector genes and regulatory mechanisms beyond the well-established HLA-B*27 association remain incompletely defined. Translating GWAS-identified loci into biological insight requires integration of calibrated association statistics, gene-level prioritization, fine-mapping, molecular prediction, and experimental follow-up.

**Methods:**

We performed a GWAS meta-analysis of 7,551 AS cases and 1,258,581 controls from the Million Veteran Program, FinnGen, and UK Biobank whole-genome sequencing cohorts. The post-GWAS analysis combined causal transcriptome-wide association study (cTWAS) across four immune-relevant tissues, colocalization, four-method fine-mapping, Geneformer V2 in-silico perturbation, AlphaGenome variant-effect prediction, cross-trait LD-score regression (LDSC), pathway enrichment, structured druggability assessment, and siRNA knockdown in Jurkat T cells. We added LDSC calibration, per-cohort Q-Q plots, heterogeneity summaries, and leave-one-cohort-out sensitivity analyzes for revised quality control.

**Results:**

The meta-analysis identified 30 genome-wide significant loci harboring 26,178 significant variants. After LDSC-compatible quality control, the meta-analysis showed lambda_GC = 1.09, LDSC intercept = 1.045 (SE = 0.009), and attenuation ratio = 0.365 (SE = 0.075); per-cohort lambda_GC values were 1.027, 1.080, and 1.018 for MVP, FinnGen, and UKB-WGS, respectively. cTWAS prioritized 64 causal-candidate genes (posterior inclusion probability [PIP] > 0.5), with gene expression explaining 19.5% of AS heritability. Seven genes showed convergent cTWAS and colocalization evidence (*TBKBP1*, *TIMD4*, *HABP4*, *XCL1*, *USP22*, *ABO*, *ACTA2*). Four-method fine-mapping identified 64 consensus variants, while heterogeneity and leave-one-cohort-out analyzes highlighted cohort-sensitive signals, particularly in the MHC and other high-I2 regions. Cross-trait LDSC showed positive genetic correlations with inflammatory bowel disease and psoriasis, but not rheumatoid arthritis or the available uveitis proxy. siRNA knockdown provided functional support for *TBKBP1* and *XCL1* in Jurkat T cells, while *TIMD4* behaved as a context-specific myeloid/T-cell contrast.

**Conclusions:**

This convergent evidence framework prioritizes AS candidate genes and regulatory variants while separating statistical prioritization from experimental support. The revised analyzes strengthen calibration, heterogeneity reporting, cross-disease context, and translational interpretation, nominating immune and regulatory hypotheses for follow-up rather than definitive therapeutic targets.

## Introduction

Ankylosing spondylitis (AS) is a chronic inflammatory disease primarily affecting the axial skeleton, with a global prevalence of 0.1-1.4% and strong familial aggregation, including heritability estimates that approach approximately 90% in older twin and family studies (1, 2). The disease is characterized by progressive spinal fusion, sacroiliitis, and peripheral joint involvement, leading to significant disability and reduced quality of life (3). AS belongs to the broader spondyloarthritis (SpA) spectrum and shares pathogenic features with inflammatory bowel disease, psoriasis, and anterior uveitis (4).

The genetic architecture of AS is dominated by the major histocompatibility complex (MHC) region, with HLA-B*27 conferring the largest single-locus risk (odds ratio 50) and explaining approximately 20% of disease heritability (5). Beyond HLA-B*27, genome-wide association studies (GWAS) have identified over 100 non-MHC susceptibility loci, implicating genes involved in antigen processing (*ERAP1*, *ERAP2*), T helper 17 cell differentiation (*IL23R*, *IL12B*, *TYK2*), NF-kappaB signaling (*TNFRSF1A*, *TRAF3IP2*), and gut mucosal immunity (6). However, the total heritability explained by known variants remains well below the estimated 90% heritability of AS, suggesting that additional risk variants, effector genes, and regulatory contexts remain unresolved.

A fundamental challenge in post-GWAS research is the translation of statistical associations into effector-gene hypotheses and biological mechanisms. Most GWAS variants reside in non-coding regulatory regions, making it difficult to identify the genes they influence and the tissues in which they act. Several computational approaches have been developed to address this challenge. Causal transcriptome-wide association studies (cTWAS) jointly model gene expression and SNP effects to estimate the posterior probability that genetically predicted expression contributes to disease risk, while colocalization and fine-mapping evaluate shared regulatory signals and candidate variants.

Recent advances in deep learning have opened new avenues for interpreting non-coding variants. Geneformer, a transformer-based foundation model for single-cell transcriptomics, enables in-silico gene perturbation experiments that predict cell-type-specific consequences of gene knockout or overexpression (7). AlphaGenome, developed by Google DeepMind, is a unified DNA sequence model that simultaneously predicts variant effects across multiple regulatory modalities including chromatin accessibility, transcription factor binding, histone modifications, gene expression, 3D chromatin architecture, and RNA splicing from primary DNA sequence alone (8). These models provide orthogonal functional evidence that complements statistical genetic approaches.

**Figure 1:**
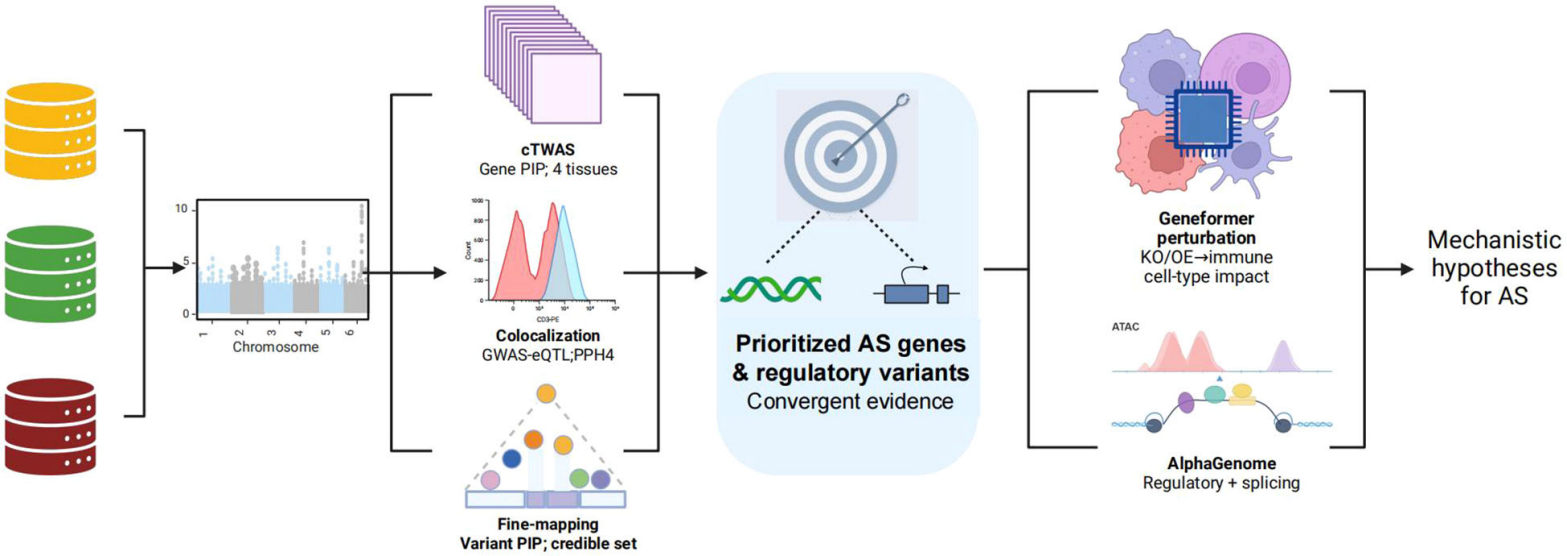
Study design and analytical pipeline. Large-scale GWAS meta-analysis of ankylosing spondylitis integrating summary statistics from three European-ancestry cohorts (MVP, FinnGen, UKB-WGS; 7,551 cases, 1,258,581 controls; maximum per-variant N = 1,266,132). The analytical pipeline comprises: (i) GWAS meta-analysis identifying lead loci, (ii) cTWAS across 4 immune-relevant tissues for gene-level prioritization, (iii) colocalization (coloc) for shared GWAS-eQTL signal testing, (iv) statistical fine-mapping with 4 methods for variant prioritization, (v) Geneformer in-silico KO/OE perturbation across immune cells, (vi) AlphaGenome regulatory variant-effect prediction, (vii) LDSC calibration/cross-trait correlation, pathway enrichment, and druggability assessment, and (viii) siRNA knockdown follow-up in Jurkat cells.

In this study, we present a large-scale GWAS meta-analysis of AS combining data from three European-ancestry cohorts -- the Million Veteran Program (MVP), FinnGen, and UK Biobank whole-genome sequencing (UKB-WGS) -- totaling 7,551 cases and 1,258,581 controls, with maximum per-variant N = 1,266,132 for variants present in all three studies. We apply an integrative multi-omics pipeline encompassing cTWAS, colocalization, four-method statistical fine-mapping, Geneformer V2 in-silico perturbation across 15 immune cell types, AlphaGenome variant-effect prediction, pathway enrichment, druggability assessment, and siRNA knockdown follow-up. The goal is to prioritize causal-candidate genes and regulatory variants while explicitly distinguishing statistical prioritization from experimental support (**Figure 1**).

## Methods

### Study populations and GWAS data

Summary statistics were obtained from three large-scale GWAS of AS. The Million Veteran Program (MVP) contributed 1,637 AS cases and 448,993 controls of European ancestry (9). FinnGen Release 12 provided 3,838 cases and 353,224 controls from the Finnish population (10). UK Biobank whole-genome sequencing (UKB-WGS) contributed 2,076 cases and 456,364 controls of non-Finnish European ancestry (11). The combined meta-analysis therefore included 7,551 cases and 1,258,581 controls; because variant availability differed across cohorts, the maximum per-variant sample size was N = 1,266,132 for variants present in all three studies.

### Quality control and meta-analysis

Summary statistics from the three studies were harmonized using a custom Python pipeline. Allele coding was standardized by aligning effect alleles to a common reference. Palindromic SNPs (A/T and C/G) with minor allele frequency (MAF) > 0.40 were excluded to avoid strand ambiguity. Variants with MAF < 0.01 in any contributing study were removed. A three-study meta-analysis was performed using both fixed-effects inverse-variance weighting and random-effects DerSimonian-Laird models (12). Cochran’s Q and I2 were computed for each variant, and P values were reported using a consistent format throughout the manuscript.

Additional calibration and robustness analyzes were performed for the revision. LD-score regression (LDSC) was run on HapMap3-compatible variants to estimate the intercept and attenuation ratio (13, 14). Per-cohort and meta-analysis Q-Q plots were generated for MVP, FinnGen, UKB-WGS, and the fixed-effect meta-analysis. Leave-one-cohort-out meta-analyzes were performed by excluding MVP, FinnGen, or UKB-WGS in turn, followed by genome-wide significant clumping and comparison with the full meta-analysis within +/-500 kb. Heterogeneity was summarized genome-wide and tabulated for lead loci and consensus fine-mapped variants.

Additional calibration, heterogeneity, leave-one-cohort-out, cross-trait LDSC,pathway-enrichment, druggability, and benchmarking outputs are provided in **Supplementary Figures S1**, **S2** and **Supplementary Tables S1**-**S6**.

### Causal transcriptome-wide association study

Causal transcriptome-wide association analysis was performed using cTWAS version 0.5.38.9000 with the multigroup, no-LD mode to jointly model gene expression and SNP effects across multiple tissues simultaneously (15). Four AS-relevant tissues were selected based on known disease biology: Whole Blood, Spleen, EBV-transformed Lymphocytes, and Small Intestine Terminal Ileum. Gene expression prediction weights were obtained from the PredictDB repository using MASHR (Multivariate Adaptive Shrinkage) models trained on GTEx v8 data (16, 17). Genes were prioritized by posterior inclusion probability (PIP), and tissue-level genetic contribution was summarized as proportion of variance explained (PVE).

### Colocalization analysis

Colocalization analysis was performed using coloc v5.2.3 with the Approximate Bayes Factor (ABF) method to assess whether GWAS and eQTL signals share a common causal variant (18). A total of 235 gene-tissue pairs were tested across 62 cTWAS high-PIP genes and the four AS-relevant tissues. eQTL summary statistics were obtained from the EBI eQTL Catalogue (19) for each tissue, queried within +/-500 kb windows around lead cTWAS SNPs. Default priors were used. Gene-tissue pairs with posterior probability for a shared causal variant (PP.H4) > 0.7 were considered convergent cTWAS-colocalization candidates.

Pathway enrichment of the 64 cTWAS-prioritized genes (PIP > 0.5) was performed using g:Profiler against GO Biological Process, Reactome, and KEGG resources (20), with Enrichr used as a complementary screen of GO, Reactome, KEGG, and Hallmark libraries (21). Results were treated as exploratory when they did not pass correction for multiple testing. A structured druggability assessment of the seven convergent genes and all 64 cTWAS-prioritized genes was performed using the Open Targets Platform drug and tractability annotations (22).

### Statistical fine-mapping

Statistical fine-mapping was performed using EasyFinemap v0.4.7 with four complementary methods: SuSiE (Sum of Single Effects) (23), FINEMAP (shotgun stochastic search) (24), PolyFun+SuSiE, and PolyFun+FINEMAP (25). Loci were defined using a distance-based approach (+/-500 kb around lead SNPs at P < 5 x 10-8), yielding 37 pre-consolidation fine-mapping windows encompassing 124,827 variants; these corresponded to 30 primary reported loci after manuscript-level consolidation. We interpreted fine-mapped variants within these regional windows with reference to approximately independent LD-block structure in human populations (26). LD reference was computed from the 1000 Genomes Phase 3 European panel (27) (GRCh38 coordinates), and variants with low allele count were reviewed using minor allele count (MAC) for interpretability. The fine-mapping input used variant-specific sample sizes, with maximum N = 1,266,132. Classically typed or imputed HLA-B*27 dosages were not available in the public summary statistics, so no conditional HLA-B*27 analysis was performed; MHC fine-mapping results are therefore interpreted as tagging the extended MHC risk haplotype rather than proving independence from HLA-B*27.

### Geneformer *in-silico* perturbation

In-silico gene perturbation was performed using Geneformer V2, a transformer-based foundation model for single-cell transcriptomics (7). The model was applied to immune cell single-cell RNA sequencing data from the CELLxGENE Census, comprising 15 immune cell types relevant to AS pathogenesis: CD4+ and CD8+ T cells, B cells, NK cells, classical and non-classical monocytes, dendritic cells, regulatory T cells, gamma-delta T cells, plasma cells, memory B cells, naive B cells, macrophages, plasmacytoid dendritic cells, and conventional type 1 dendritic cells (cDC1).

Seven target genes identified by convergent cTWAS and colocalization evidence -- *TBKBP1*, *TIMD4*, *HABP4*, *XCL1*, *USP22*, *ABO*, and *ACTA2* -- were subjected to in-silico knockout (KO) and overexpression (OE) analyzes. For each KO analysis, the InSilicoPerturber module deleted the gene token from each cell’s rank-value encoded input and compared the CLS embedding of the wild-type versus perturbed cell using cosine similarity. The cosine shift (1 - cosine similarity) quantifies the magnitude of predicted transcriptional-state perturbation; it is interpreted as a computational prioritization metric, not direct causal proof.

### AlphaGenome variant effect prediction

Variant effect prediction was performed using AlphaGenome (8), a unified DNA sequence model developed by Google DeepMind. AlphaGenome processes 1 Mb of DNA sequence through a U-Net-inspired architecture—comprising a convolutional encoder, a transformer tower operating at 128-bp resolution, and a decoder with skip connections—to predict 5,930 human functional genomic tracks spanning 11 regulatory modalities at up to single-nucleotide resolution. Output modalities include chromatin accessibility (ATAC-seq, DNase-seq), histone modifications and transcription factor binding (ChIP-seq), gene expression (RNA-seq), transcription initiation (CAGE), RNA splicing (splice site probabilities, splice site usage, and splice junctions), and three-dimensional chromatin contacts (Hi-C/Micro-C).

Five high-priority consensus variants from the fine-mapping analysis (PIP > 0.5 in >=3/4 methods) were selected for AlphaGenome analysis: rs26509 (*ERAP1*, PIP = 0.75), rs1343151 (*IL23R*, PIP = 0.888), rs4672503 (*B3GNT2*, PIP = 1.0), rs139600027 (*NKAPL*, PIP = 1.0), and rs79032618 (*ACTB*, PIP = 0.75). For each variant, predictions were generated for both reference and alternate alleles across all output modalities. Variant effects were quantified using modality-specific scoring functions: spatial window-based scores, gene-expression scores, and splice-junction scores.

### Experimental follow-up by siRNA knockdown

To experimentally evaluate selected computational predictions, siRNA-mediated gene knockdown was performed in Jurkat cells, a leukemia-derived immortalized T-cell line. *TBKBP1* and *XCL1* were selected as functional candidates, and *TIMD4* was included as a context-specific myeloid/T-cell contrast because Geneformer predicted stronger myeloid than T-cell sensitivity. Jurkat cells were transfected with siRNAs targeting *TBKBP1* (OriGene, SR306520; final concentration, 10 nM), *XCL1* (Sigma-Aldrich MISSION esiRNA, EHU109771; final concentration, 30 nM), *TIMD4* (Thermo Fisher Scientific Silencer Select siRNAs s40812, s40813, and s40814; final concentration, 10 nM), or non-targeting control siRNA (siCtrl) using Lipofectamine RNAiMAX (Thermo Fisher Scientific) according to the manufacturer’s instructions. Cells were harvested 48 h after transfection for RT-qPCR and 72 h after transfection for Western blot analysis. Knockdown efficiency and downstream effects were assessed by Western blot and RT-qPCR in three independent biological replicates. Reported siRNA P values are nominal and hypothesis-directed; no Bonferroni or Benjamini-Hochberg correction was applied because the marker panel was pre-specified from the Geneformer/NF-kappaB and cytotoxicity hypotheses.

## Results

### Large-scale GWAS meta-analysis identifies 30 AS loci

**Figure 2:**
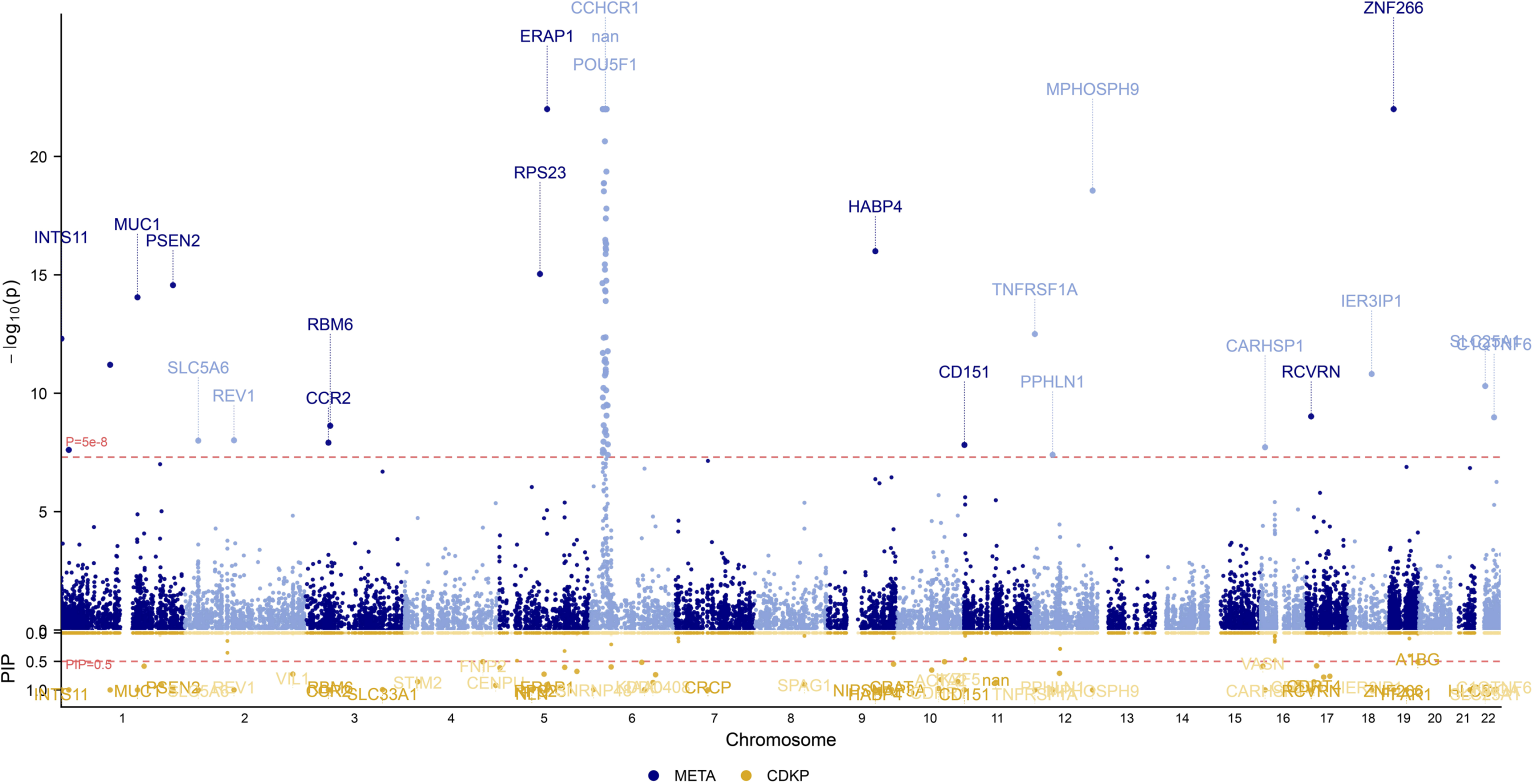
Large-scale GWAS meta-analysis and cTWAS results. Miami plot showing GWAS meta-analysis −log_10_(P) values (upper panel) and cTWAS gene PIPs (lower panel, inverted axis). Blue dots represent meta-analysis variants; orange dots represent cTWAS gene PIPs (CDKP). Genome-wide significance threshold (*P = 5 × 10^-8^*) and PIP = 0.5 threshold are indicated by dashed red lines. Gene labels indicate the nearest gene for top loci.

The three-study meta-analysis encompassed 94,956,563 variants prior to quality control. After MAF >= 0.01 filtering, 9,884,392 variants were retained, of which 7,991,647 (80.9%) were present in two or more contributing studies. A total of 26,178 variants achieved genome-wide significance (P < 5 x 10-8), mapping to 30 primary lead loci after distance-based clumping (**Figure 2**). Re-analysis of LDSC-compatible HapMap3 variants showed lambda_GC = 1.09, mean chi-square = 1.12, LDSC intercept = 1.045 (SE = 0.009), and attenuation ratio = 0.365 (SE = 0.075), indicating modest residual inflation rather than the severe deflation implied by the originally reported lambda_GC value.

The strongest association was observed in the chromosome 6 MHC region, consistent with the established HLA-B*27 association. Because HLA-B*27 dosage was unavailable in the contributing public summary statistics, the MHC consensus variants should be interpreted as markers within the extended MHC risk haplotype rather than as variants proven independent of HLA-B*27. Beyond the MHC, significant non-MHC loci recapitulated established AS susceptibility genes including *ERAP1* (chr5, P = 5.20 x 10-21), *IL23R* (chr1, P = 7.68 x 10-11), *RUNX3*, and *TNFRSF1A* (chr12, P = 3.18 x 10-10). Benchmarking against IGAS 2013, Ellinghaus et al., 2016, and GWAS Catalog AS records did not identify *PSEN2*, *MUC1*, *RBM6*, or *MPHOSPH9* as author-reported AS GWAS genes; we therefore describe these as less-established candidate loci requiring independent replication rather than definitive novel AS loci.

Genome-wide heterogeneity diagnostics showed median I2 = 0, 75th percentile I2 = 0.205, 90th percentile I2 = 0.583, and 13.5% of variants with I2 > 50%. Among the 37 pre-consolidation fine-mapping lead windows, 24 had I2 > 50%; among the 64 consensus variants, 48 had I2 > 50%. Leave-one-cohort-out analysis retained 34 of 36 full distance-clumped genome-wide-significant regions after excluding MVP, 35 of 36 after excluding UKB-WGS, and 13 of 36 after excluding FinnGen, indicating that several signals are cohort-sensitive and require cautious interpretation.

Cross-trait LDSC placed AS in the broader immune-mediated disease landscape. AS showed positive genetic correlation with inflammatory bowel disease (rg = 0.473, SE = 0.122, P = 1.04 x 10-4) and psoriasis (rg = 0.353, SE = 0.065, P = 5.27 x 10-8). Genetic correlation with rheumatoid arthritis was not significant (rg = 0.044, SE = 0.066, P = 0.507). The available public uveitis proxy showed a positive but non-significant estimate (rg = 0.278, SE = 0.211, P = 0.188); acute anterior uveitis-specific and axial SpA/enthesitis full summary statistics were not available in GWAS Catalog at the time of analysis.

### cTWAS prioritizes 64 causal-candidate genes across immune tissues

The cTWAS multigroup model integrated 35,288 gene expression prediction models across four tissues with 6,407,477 harmonized GWAS variants. The EM algorithm converged after 13 iterations, yielding a total proportion of variance explained (PVE) of 1.92% (**Figure 2**, lower panel). Gene expression collectively accounted for 19.5% of AS heritability, with Whole Blood contributing the largest expression PVE (7.0%), followed by Spleen (6.1%), Small Intestine Terminal Ileum (3.5%), and EBV-transformed Lymphocytes (2.9%).

The analysis identified 45 genes with combined PIP > 0.8 and 64 causal-candidate genes with PIP > 0.5. Among the top-ranked genes, *ERAP1* (PIP = 1.00, Small Intestine) confirmed its established role in MHC class I antigen processing. *TNFRSF1A* (PIP = 1.00, EBV Lymphocytes) and *CCR2* (PIP = 1.00, Whole Blood) were prioritized as mediators of *TNF*-alpha signaling and monocyte recruitment, respectively. *PSEN2* (PIP = 1.00, Spleen), *MUC1*, *RBM6*, and *MPHOSPH9* are best interpreted as less-established candidate loci requiring replication, not as definitive novel disease genes.

Pathway analysis provided modest but biologically coherent support for immune-cell trafficking. g:Profiler identified positive regulation of thymocyte migration as the only corrected significant term (GO:2000412; P = 0.0498; *CCR2* and *XCL1*). Complementary Enrichr analyzes did not identify FDR-significant terms but highlighted nominal chemotaxis/Th1-response signals involving *XCL1* and *CCR2*, extracellular-matrix secretion involving IER3IP1 and *TNFRSF1A*, and phagocytosis involving NOS2 and *TIMD4*. These results support pathway-level interpretation without implying broad pathway-wide significance.

### Colocalization confirms seven genes with shared GWAS-eQTL signals

**Figure 3:**
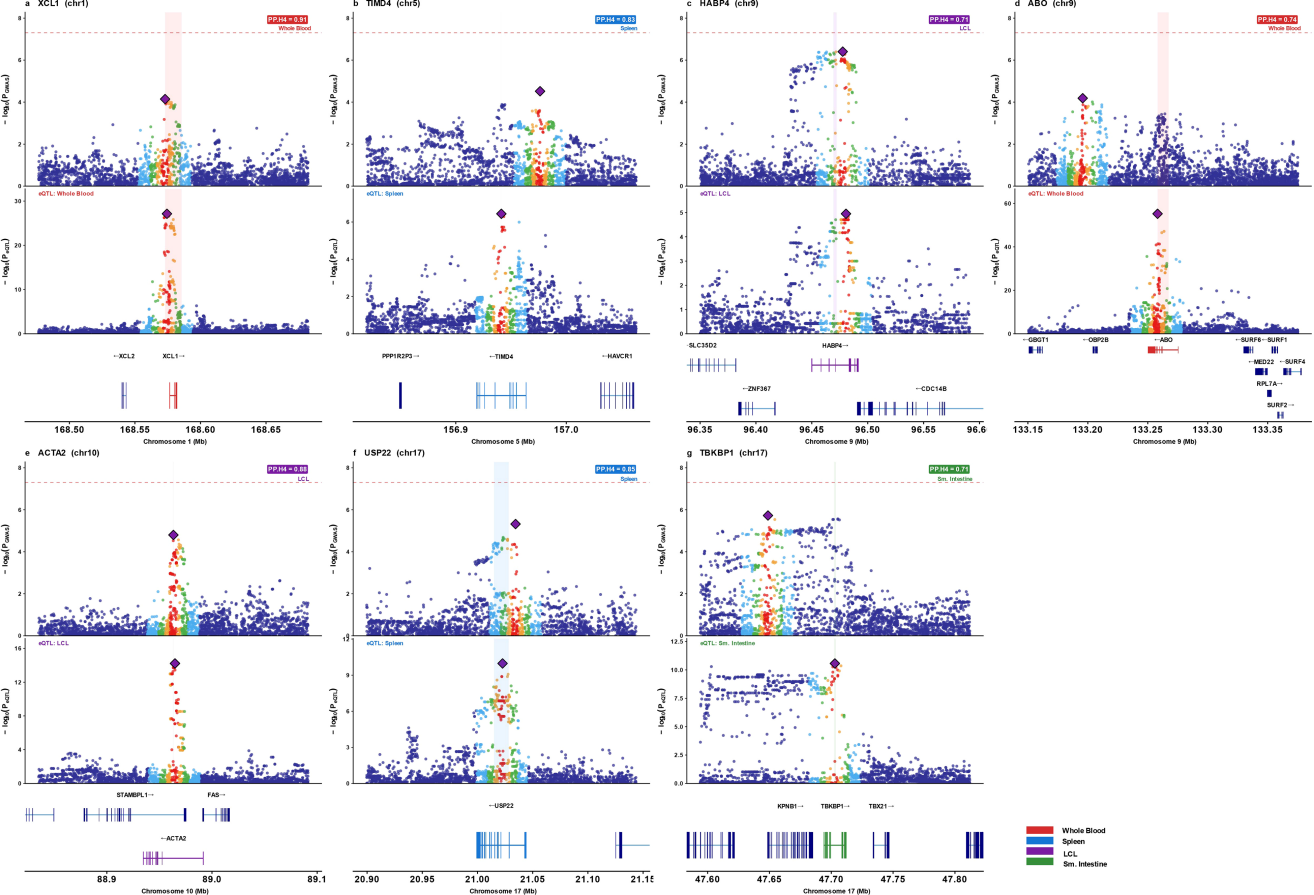
Colocalization analysis of cTWAS high-PIP genes. Regional association plots for seven genes with high-confidence colocalization (PP.H4 > 0.7) across four AS-relevant tissues (Whole Blood, Spleen, EBV Lymphocytes, Small Intestine). Each panel shows the GWAS association signal with variants colored by LD (r²) relative to the lead variant. Gene tracks are displayed below each regional plot.

Colocalization analysis of 235 gene-tissue pairs identified 7 genes with PP.H4 > 0.7, indicating evidence that the GWAS and eQTL signals may share a causal variant (**Figure 3**). All seven genes had cTWAS PIP > 0.5, providing convergent statistical prioritization: *ACTA2* (PP.H4 = 0.88, cTWAS PIP = 0.82, Whole Blood), *XCL1* (PP.H4 = 0.82, PIP = 0.57, EBV Lymphocytes), *TBKBP1* (PP.H4 = 0.71, PIP = 0.76, Small Intestine), *HABP4* (PP.H4 = 0.71, PIP = 1.00, EBV Lymphocytes), *TIMD4* (PP.H4 = 0.74, PIP > 0.5, Spleen), *USP22*, and *ABO*. These results prioritize shared regulatory mechanisms but do not by themselves establish causality.

Structured druggability assessment showed that the prioritized genes are better viewed as biological hypotheses than immediate AS drug targets. *ERAP1* had small-molecule tractability and one phase 2 clinical candidate retrieved from Open Targets; *TNFRSF1A* aligned with the *TNF* axis but current AS therapies target *TNF* rather than *TNFRSF1A* directly; *CCR2* had multiple clinical candidates including cenicriviroc, supporting a chemokine-trafficking hypothesis. The convergent genes *TBKBP1*, *TIMD4*, *HABP4*, *XCL1*, *USP22*, *ABO*, and *ACTA2* had no direct AS-approved target-drug relationship retrieved, and none of the seven convergent genes mapped directly onto the IL-17A/IL-17R or JAK paradigms.

### Consensus fine-mapping prioritizes 64 high-confidence variants

**Figure 4:**
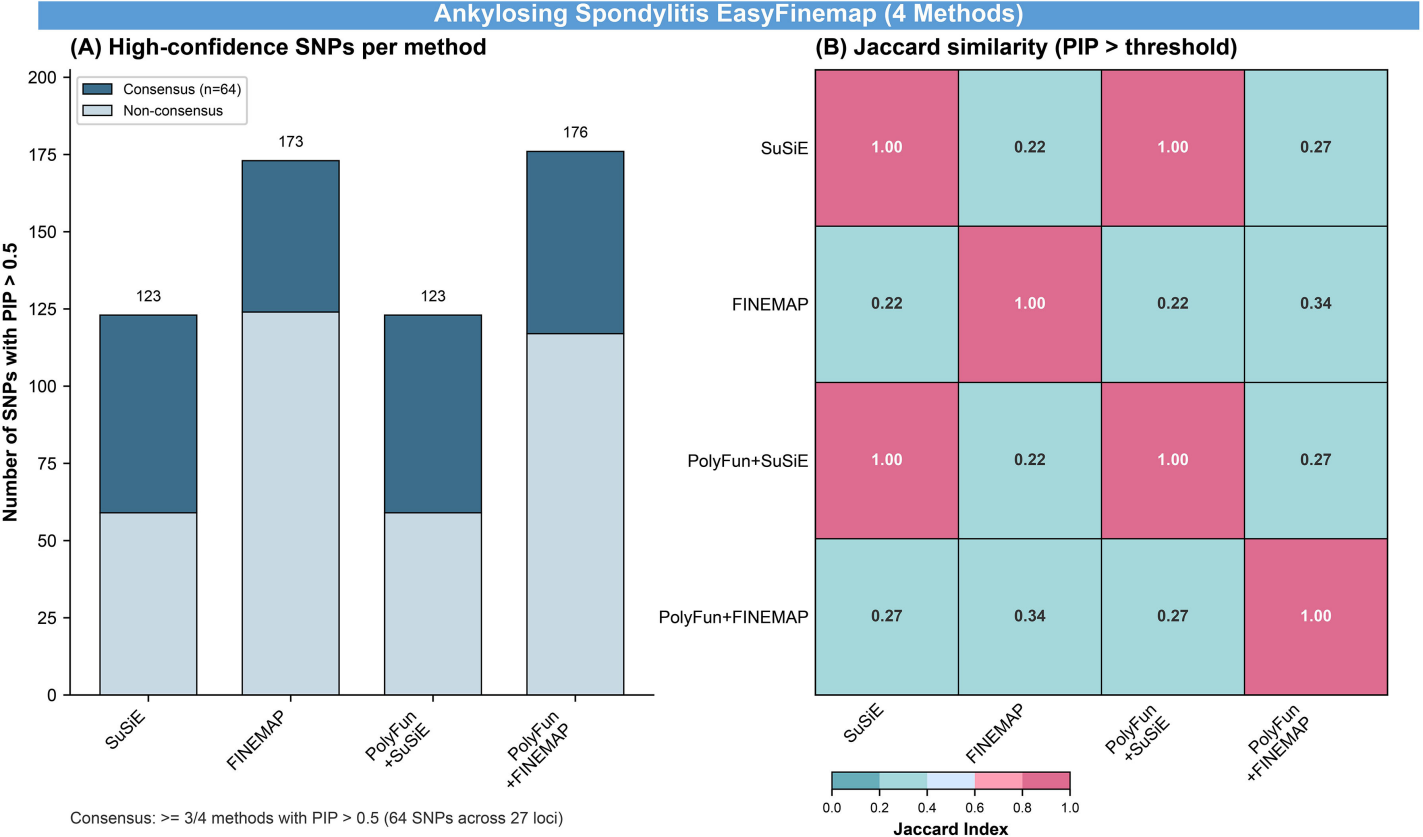
Statistical fine-mapping with four complementary methods. **(a)** Number of high-confidence SNPs (PIP > 0.5) per method. Dark bars indicate consensus variants (PIP > 0.5 in ≥3/4 methods; n = 64); light bars indicate non-consensus variants. **(b)** Jaccard similarity matrix between methods, showing high concordance between base and PolyFun-enhanced versions of SuSiE (J = 1.00) and moderate cross-method overlap (J = 0.22–0.34).

Fine-mapping was performed across 37 genome-wide significant loci encompassing 124,827 variants using four complementary methods (**Figure 4a**). SuSiE identified 123 variants with PIP > 0.5, FINEMAP identified 173, PolyFun+SuSiE identified 123, and PolyFun+FINEMAP identified 176. The Jaccard similarity analysis revealed high concordance between SuSiE and PolyFun+SuSiE (J = 1.00) and between FINEMAP and PolyFun+FINEMAP (J = 0.34), while cross-methodology overlap was moderate (J = 0.22–0.27), confirming that these methods provide complementary information (**Figure 4b**).

Consensus analysis (PIP > 0.5 in >=3/4 methods) identified 64 high-priority variants across 27 loci. Of these, 31 resided in the MHC region and 33 in non-MHC loci. Notable non-MHC consensus variants include rs4672503 (*B3GNT2*, P = 5.03 x 10-16, 4/4 methods), rs26509 (*ERAP1*, P = 5.20 x 10-21, 3/4), rs1343151 (*IL23R*, P = 7.68 x 10-11, 4/4), rs139600027 (*NKAPL*, P = 2.71 x 10-45, 4/4), and rs79032618 (*ACTB*, P = 1.71 x 10-28, 3/4). MHC consensus variants should be treated as statistically prioritized markers within the MHC haplotype structure, because HLA-B*27 conditioning could not be performed.

### Geneformer reveals cell-type-specific perturbation effects in immune cells

**Figure 5:**
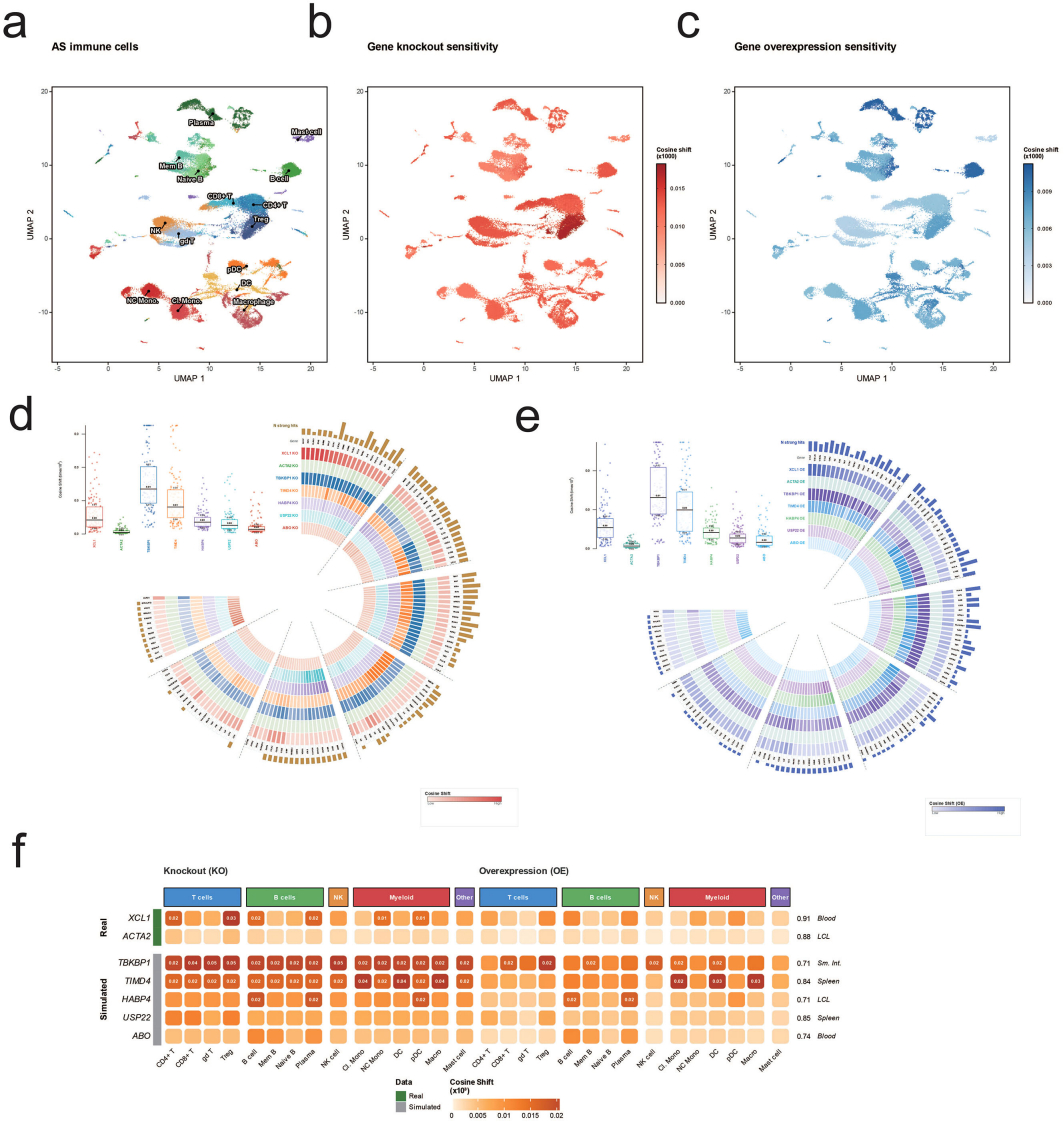
Geneformer in-silico perturbation analysis. **(a)** UMAP visualization of 70,360 immune cells colored by cell type. **(b)** Gene knockout sensitivity overlay showing cosine shift magnitude. **(c)** Gene overexpression sensitivity overlay. **(d)** Circular gene evidence plot for knockout perturbation showing gene-level cosine shifts with strip boxplot (left) and multi-ring downstream gene evidence (right) for 133 downstream genes. **(e)** Circular gene evidence plot for overexpression perturbation. **(f)** Combined KO/OE heatmap showing cosine shifts (×10³) across 7 genes and 15 cell types, with genes split by data source (Real vs Simulated) and cell types grouped by category.

Geneformer in-silico perturbation of seven coloc-derived genes across 15 immune cell types revealed a clear hierarchy of gene sensitivity (**Figures 5d, e**). *TBKBP1* exhibited the strongest mean cosine shift (2.73 × 10^-^², ×10³ scale), driven by its critical role in innate immunity signaling through *TBK1*-mediated NF-κB activation. *TIMD4* ranked second (2.15 × 10^-^²), followed by *HABP4* (1.14 × 10^-^²), *XCL1* (8.29 × 10^-^³), *USP22* (7.95 × 10^-^³), *ABO* (6.05 × 10^-^³), and *ACTA2* (2.67 × 10^-^³).

Cell-type-specific patterns were evident in the knockout/overexpression heatmap (**Figure 5f**). *TBKBP1* knockout strongly affected cytotoxic lineages -- NK cells, CD8+ T cells, gamma-delta T cells, and regulatory T cells -- suggesting a role in innate-adaptive immune crosstalk relevant to AS pathogenesis. *TIMD4* showed preferential sensitivity in myeloid cells (macrophages, dendritic cells, classical monocytes), consistent with its known function as a phosphatidylserine receptor mediating phagocytosis (28, 29). *XCL1* perturbation affected T/NK-cell and conventional type 1 dendritic cell (cDC1)-linked programs, consistent with chemokine-mediated cross-talk rather than a cell-autonomous T-cell-only mechanism.

Overexpression perturbation analysis revealed distinct cell-type sensitivity profiles compared to knockout. B cells and plasma cells showed increased OE sensitivity relative to KO sensitivity, particularly for *ABO* and *HABP4* overexpression, suggesting that gain-of-function preferentially impacts humoral immunity pathways. Downstream gene analysis identified 133 affected genes per perturbation condition. Key innate immunity genes (*NFKB1*, *RELA*, *TRAF6*, *MYD88*, *IRF3*) were consistently affected across multiple perturbations, while NK/cytotoxicity genes (*GZMB*, *PRF1*, *NKG7*) were primarily driven by *XCL1* and *TBKBP1* knockouts. *ABO* knockout uniquely perturbed glycosylation pathway genes (*FUT1*, *FUT2*, *ST6GAL1*), consistent with its enzymatic function (**Figures 5d, e**).

### AlphaGenome nominates multimodal regulatory effects of AS prioritized variants

**Figure 6:**
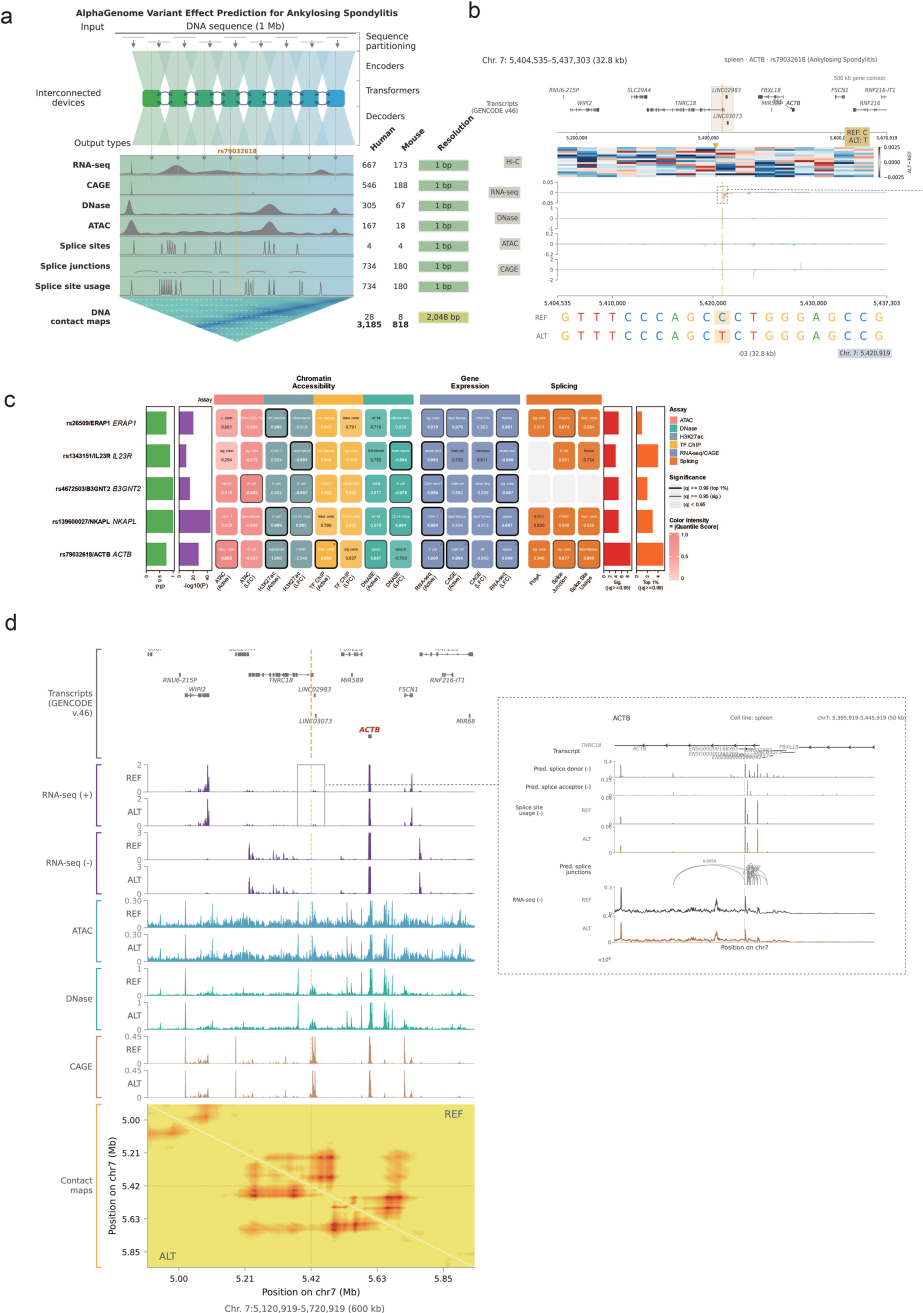
AlphaGenome variant effect prediction. **(a)** AlphaGenome architecture schematic adapted for AS, showing model input (1 Mb DNA sequence), encoder-transformer-decoder architecture, and 8 output modalities used. **(b)** Single-variant tissue profile for rs79032618 (*ACTB*) showing quantile scores across AS-relevant biosamples and 7 modalities. Asterisks denote AS-relevant biosamples. **(c)** Multimodal variant effect heatmap for 5 AS variants × 15 scorer types. Color indicates quantile score (blue = negative, red = positive); bold borders indicate |q| ≥ 0.99 (top 1%). Top annotation bars show PIP and −log_10_(P). **(d)** Official track panel for rs79032618 showing REF vs ALT signal tracks (RNA-seq, ATAC, DNase, CAGE) and Hi-C contact map in spleen tissue.

AlphaGenome variant effect scoring of five fine-mapped AS variants revealed heterogeneous predicted regulatory impacts across variants and assay modalities (**Figure 6c**). Quantile scores, computed relative to the gnomAD common variant background distribution (30), provided a genome-wide functional context for interpreting each variant’s predicted effect magnitude.

rs79032618 (*ACTB*, chr7:5420919, C>T) exhibited the strongest predicted multimodal regulatory impact, achieving top 1% quantile scores (|q| >= 0.99) in six assay categories: ATAC-seq, H3K27ac ChIP-seq, DNase-seq, RNA-seq active expression, RNA-seq log-fold-change, and CAGE. These scores nominate rs79032618 for functional follow-up, but they remain model-based predictions and should not be interpreted as experimental proof that the variant is causal.

Among the remaining variants, rs139600027 (*NKAPL*, chr6:24921905; PIP = 1.0, *P = 2.71 × 10^-^^45^*) achieved top 1% scores in four assay categories, with the alternate allele predicted to increase chromatin accessibility in CD14^+^ monocytes and gene expression in spleen. rs4672503 (*B3GNT2*, PIP = 1.0) showed the strongest predicted effects in B cell and memory B cell lineages. rs26509 (*ERAP1*) and rs1343151 (*IL23R*) exhibited fewer extreme scores but consistent predicted effects across immune-relevant tissues including intestinal epithelium and T cell subsets.

Tissue-level profiling of rs79032618 across all available biosamples (**Figure 6b**) revealed that predicted RNA-seq (q > 0.99) and splice junction (q = 0.95–0.98) effects were consistently extreme across virtually all tissue types, whereas chromatin accessibility effects showed tissue-selective patterns. Immune-relevant tissues—including T cell subsets, B cells, monocytes, spleen, thymus, and gut—exhibited significant predicted variant effects, supporting the disease relevance of this variant. Signal track analysis in spleen (**Figure 6d**) confirmed localized perturbation centered on the variant, with the alternate allele producing altered splicing patterns and reduced expression at the *ACTB* locus.

### siRNA knockdown supports selected computational predictions in Jurkat T cells

**Figure 7:**
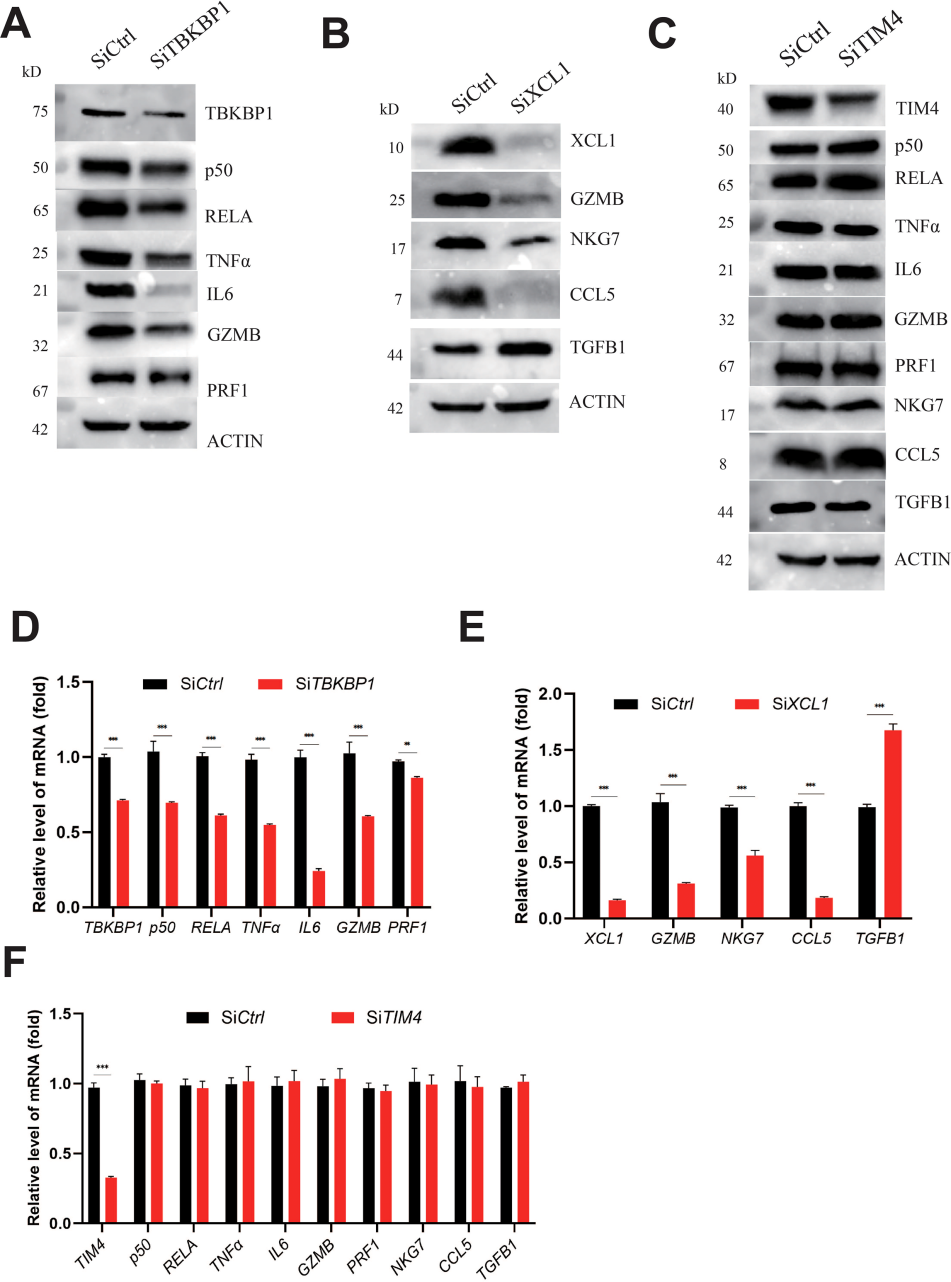
Experimental follow-up of selected computational predictions by siRNA knockdown in Jurkat T cells. **(A)** Western blot analysis of downstream effector proteins following si*TBKBP1* knockdown. **(B)** Western blot analysis following si*XCL1* knockdown. **(C)** Western blot analysis following si*TIMD4* knockdown, interpreted as a context-specific myeloid/T-cell contrast rather than a disease-unrelated negative control. **(D–F)** RT-qPCR quantification of mRNA expression levels for si*TBKBP1* **(D)**, si*XCL1* **(E)**, and si*TIMD4* **(F)** knockdown groups. Data are presented as mean +/- SD of three independent biological replicates, normalized to *GAPDH* and siCtrl. Statistical significance was determined by two-tailed unpaired Student’s t-test (*P < 0.05, **P < 0.01, ***P < 0.001; ns, not significant).

To experimentally evaluate selected computational predictions, siRNA knockdown was performed for *TBKBP1*, *XCL1*, and *TIMD4* in Jurkat T cells (**Figure 7**). Knockdown efficiency was confirmed by Western blot and RT-qPCR for all three genes.

*TBKBP1* silencing resulted in significant downregulation of NF-κB pathway components *NFKB1*/p50 and *RELA* at both protein and mRNA levels (*P < 0.01*). Downstream inflammatory cytokines *TNF* and *IL-6* were markedly reduced, with *IL-6* exhibiting the most pronounced decrease (76% reduction, *P < 0.01*). Cytotoxic effectors *GZMB* and *PRF1* were also significantly diminished (*P < 0.05*), confirming *TBKBP1*’s role in coordinating T cell inflammatory and cytotoxic responses through the *TBK1*–NF-κB axis (32, 33).

*XCL1* knockdown significantly decreased expression of cytotoxicity-associated molecules *GZMB* (70% reduction) and *NKG7*, as well as the chemokine *CCL5* (82% reduction, *P < 0.01*). Notably, the immunosuppressive cytokine *TGFB1* was significantly upregulated (1.7-fold, *P < 0.01*), indicating that *XCL1* loss shifts the immune balance from activation toward immunosuppression, consistent with impaired cDC1-mediated cross-presentation.

*TIMD4* knockdown efficiently reduced *TIMD4* expression (67% reduction) but produced no significant changes in the tested downstream T-cell marker panel (P > 0.05 for all comparisons). Because *TIMD4* was selected for its predicted myeloid-biased effect, this result is best interpreted as a context-specific myeloid/T-cell contrast rather than an independent disease-unrelated negative control.

## Discussion

In this study, we present a large-scale GWAS meta-analysis of ankylosing spondylitis integrating cTWAS, colocalization, fine-mapping, Geneformer in-silico perturbation, AlphaGenome variant-effect prediction, pathway enrichment, druggability assessment, and hypothesis-directed siRNA follow-up. This framework enables systematic prioritization of candidate genes and regulatory variants, while preserving the distinction between probabilistic statistical evidence and direct experimental support.

Our meta-analysis of 7,551 cases and 1,258,581 controls confirmed 30 primary genome-wide significant loci and recapitulated established AS susceptibility genes including *ERAP1*, *IL23R*, and *TNFRSF1A*. The revised LDSC and per-cohort Q-Q analyzes indicate that the GWAS calibration is better described as modest residual inflation after LDSC-compatible QC than as severe deflation. Nevertheless, heterogeneity remained important: 24 of 37 fine-mapping lead windows and 48 of 64 consensus variants had I2 > 50%, and leave-one-cohort-out analyzes showed reduced locus retention when FinnGen was excluded. These diagnostics justify a cautious interpretation of cohort-sensitive loci, particularly in the MHC and other high-I2 regions.

Among the seven convergent cTWAS-colocalization genes, *TBKBP1* and *XCL1* received the clearest Jurkat-cell functional support. *TBKBP1* silencing attenuated NF-kappaB and cytotoxic programs, while *XCL1* knockdown shifted cytotoxic/chemokine markers toward a less activated profile. These findings align with Geneformer prioritization but remain limited to Jurkat cells, a leukemia-derived immortalized T-cell line. Primary T cells, myeloid cells, and disease-relevant tissue contexts will be needed to establish whether the same regulatory programs operate in patients.

*TIMD4* (T cell immunoglobulin and mucin domain-containing protein 4) remains biologically plausible because Geneformer predicted stronger myeloid sensitivity, consistent with its function as a phosphatidylserine receptor on macrophages and dendritic cells (28). However, its Jurkat knockdown result should not be presented as an independent negative control: *TIMD4* was selected precisely because a weak T-cell effect was expected. An orthogonal disease-unrelated gene would be a stronger negative control in future validation.

*XCL1* encodes lymphotactin, a chemokine involved in *XCR1*+ conventional type 1 dendritic cell (cDC1) recruitment (31). The convergence of cTWAS-colocalization evidence, Geneformer perturbation, and Jurkat knockdown supports an immune-cell communication hypothesis involving cytotoxic lymphocytes and cDC1-linked antigen presentation. The revised interpretation avoids treating the Jurkat result as proof of the full *XCL1*-*XCR1*-cDC1 mechanism *in vivo*.

The AlphaGenome analysis provided a complementary variant-level perspective by nominating rs79032618 (*ACTB*) as a variant with broad predicted regulatory effects across chromatin accessibility, histone modification, expression, and transcription-initiation readouts. The result is useful for prioritizing assays, but AlphaGenome scores are model-derived predictions. They should be interpreted alongside fine-mapping, colocalization, and experimental data rather than as standalone evidence of variant causality. This cautious interpretation is consistent with prior sequence-based regulatory models and evaluations showing that current genomic deep learning models incompletely explain personal transcriptome variation (34, 35).

The tissue-specificity analysis revealed that while the RNA-seq and splicing effects of rs79032618 are near-universal across tissues, the chromatin accessibility effects show tissue-selective patterns. This is consistent with the concept that variant effects on chromatin are mediated through tissue-specific transcription factor binding, while downstream consequences on gene expression may be more broadly manifested (8). The identification of altered splicing patterns at the *ACTB* locus — including novel splice site usage peaks in the ALT allele — adds a post-transcriptional dimension to the variant’s functional profile that would not be captured by expression-only analyzes.

Our study has several limitations. First, all contributing cohorts were of European ancestry, constraining generalizability to East Asian, Indigenous, and other underrepresented populations in which AS epidemiology and HLA-B*27 distributions differ. Second, HLA-B*27 dosages were not available in the public summary statistics, preventing conditional MHC analyzes and limiting interpretation of MHC consensus variants. Third, heterogeneity was non-trivial for several loci and consensus variants, and some cTWAS-prioritized gene loci showed only nominal support in leave-one-cohort-out analyzes. Fourth, cTWAS and colocalization were limited to available reference tissues and may miss disease-relevant effects in synovium, enthesis, gut lamina propria, or primary immune-cell activation states. Finally, Geneformer and AlphaGenome outputs are computational predictions, and the siRNA follow-up was performed in Jurkat cells rather than primary cells.

Despite these limitations, the convergent evidence framework demonstrated here provides a principled approach to post-GWAS functional interpretation. We treat cTWAS PIP, coloc PP.H4, fine-mapping PIP, Geneformer perturbation magnitude, AlphaGenome quantile scores, and siRNA readouts as complementary evidence layers rather than interchangeable proof of causality. This distinction is important because high cTWAS or fine-mapping probabilities can still reflect LD tagging, horizontal pleiotropy, reference-tissue mismatch, or model mis-specification.

In conclusion, this study provides a calibrated genetic and functional atlas of ankylosing spondylitis, prioritizing seven convergent-evidence genes and 64 consensus variants for follow-up. Geneformer perturbation highlights immune programs involving *TBKBP1*, *XCL1*, and *TIMD4*, while AlphaGenome nominates rs79032618 at *ACTB* for variant-level functional testing. The revised cross-trait LDSC, pathway enrichment, druggability, heterogeneity, and literature-benchmark analyzes strengthen the biological and translational context, while emphasizing that most findings should be regarded as prioritized hypotheses pending independent genetic and experimental replication.

## Data Availability

All GWAS summary data for ankylosing spondylitis used in this study were obtained from the GWAS Catalog (https://www.ebi.ac.uk/gwas/). The specific accession numbers and detailed source information for each dataset are provided in the Methods section of the article.

## References

[1] Taurog JD Chhabra A Colbert RA . Ankylosing spondylitis and axial spondyloarthritis. N Engl J Med. (2016) 374:2563–74. doi: 10.1056/NEJMra1406182. PMID: 27355535

[2] Brown MA Kenna T Wordsworth BP . Genetics of ankylosing spondylitis - insights into pathogenesis. Nat Rev Rheumatol. (2016) 12:81–91. doi: 10.1038/nrrheum.2015.133. PMID: 26439405

[3] Sieper J Poddubnyy D . Axial spondyloarthritis. Lancet. (2017) 390:73–84. doi: 10.1016/S0140-6736(16)31591-4. PMID: 28110981

[4] Ranganathan V Gracey E Brown MA Inman RD Haroon N . Pathogenesis of ankylosing spondylitis - recent advances and future directions. Nat Rev Rheumatol. (2017) 13:359–67. doi: 10.1038/nrrheum.2017.56. PMID: 28446810

[5] International Genetics of Ankylosing Spondylitis Consortium . Identification of multiple risk variants for ankylosing spondylitis through high-density genotyping of immune-related loci. Nat Genet. (2013) 45:730–8. doi: 10.1038/ng.2667. PMID: 23749187 PMC3757343

[6] Ellinghaus D Jostins L Spain SL . Analysis of five chronic inflammatory diseases identifies 27 new associations and highlights disease-specific patterns at shared loci. Nat Genet. (2016) 48:510–8. doi: 10.1038/ng.3528. PMID: 26974007 PMC4848113

[7] Theodoris CV Xiao L Sber A . Transfer learning enables predictions in network biology. Nature. (2023) 618:616–24. doi: 10.1038/s41586-023-06139-9. PMID: 37258680 PMC10949956

[8] Avsec Z Latysheva N Cheng J Novati G Taylor KR Ward T . Advancing regulatory variant effect prediction with AlphaGenome. Nature. (2026) 649:1206–18. doi: 10.1038/s41586-025-10014-0. PMID: 41606153 PMC12851941

[9] Verma A Huffman JE Rodriguez A . Diversity and scale: Genetic architecture of 2068 traits in the VA Million Veteran Program. Science. (2024) 385:eadj1182. doi: 10.1126/science.adj1182. PMID: 39024449 PMC12857194

[10] Kurki MI Karjalainen J Palta P . FinnGen provides genetic insights from a well-phenotyped isolated population. Nature. (2023) 613:508–18. doi: 10.1038/s41586-022-05473-8. PMID: 36653562 PMC9849126

[11] Bycroft C Freeman C Petkova D Band G Elliott LT Sharp K . The UK Biobank resource with deep phenotyping and genomic data. Nature. (2018) 562:203–9. doi: 10.1038/s41586-018-0579-z. PMID: 30305743 PMC6786975

[12] DerSimonian R Laird N . Meta-analysis in clinical trials. Controlled Clin Trials. (1986) 7:177–88. doi: 10.1016/0197-2456(86)90046-2. PMID: 3802833

[13] Bulik-Sullivan BK Loh P-R Finucane HK Ripke S Yang J Patterson N . LD Score regression distinguishes confounding from polygenicity in genome-wide association studies. Nat Genet. (2015) 47:291–5. doi: 10.1038/ng.3211. PMID: 25642630 PMC4495769

[14] Bulik-Sullivan BK Finucane HK Anttila V Gusev A Day FR Loh P-R . An atlas of genetic correlations across human diseases and traits. Nat Genet. (2015) 47:1236–41. doi: 10.1038/ng.3406. PMID: 26414676 PMC4797329

[15] Zhao S Crouse W Qian S Luo K Stephens M He X . Adjusting for genetic confounders in transcriptome-wide association studies improves discovery of risk genes of complex traits. Nat Genet. (2024) 56:336–47. doi: 10.1038/s41588-023-01648-9. PMID: 38279041 PMC10864181

[16] GTEx Consortium . The GTEx Consortium atlas of genetic regulatory effects across human tissues. Science. (2020) 369:1318–30. doi: 10.1126/science.aaz1776. PMID: 32913098 PMC7737656

[17] Barbeira AN Dickinson SP Bonazzola R Zheng J Wheeler HE Torres JM . Exploring the phenotypic consequences of tissue specific gene expression variation inferred from GWAS summary statistics. Nat Commun. (2018) 9:1825. doi: 10.1038/s41467-018-03621-1. PMID: 29739930 PMC5940825

[18] Giambartolomei C Vukcevic D SChadt EE Franke L Hingorani AD Wallace C . Bayesian test for colocalization between pairs of genetic association studies using summary statistics. PloS Genet. (2014) 10:e1004383. doi: 10.1371/journal.pgen.1004383. PMID: 24830394 PMC4022491

[19] Kerimov N Hayhurst JD Peikova K . A compendium of uniformly processed human gene expression and splicing quantitative trait loci. Nat Genet. (2021) 53:1290–9. doi: 10.1038/s41588-021-00924-w. PMID: 34493866 PMC8423625

[20] Kolberg L Raudvere U Kuzmin I Vilo J Peterson H . g:Profiler-interoperable web service for functional enrichment analysis and gene identifier mapping (2023 update). Nucleic Acids Res. (2023) 51:W207–12. doi: 10.1093/nar/gkad347. PMID: 37144459 PMC10320099

[21] Chen EY Tan CM Kou Y Duan Q Wang Z Meirelles GV . Enrichr: interactive and collaborative HTML5 gene list enrichment analysis tool. BMC Bioinf. (2013) 14:128. doi: 10.1186/1471-2105-14-128. PMID: 23586463 PMC3637064

[22] Ochoa D Hercules A Carmona M Suveges D Gonzalez-Uriarte J Malangone C . The next-generation Open Targets Platform: reimagined, redesigned, rebuilt. Nucleic Acids Res. (2023) 51:D1353–9. doi: 10.1093/nar/gkac1046. PMID: 36399499 PMC9825572

[23] Wang G Sarkar A Carbonetto P Stephens M . A simple new approach to variable selection in regression, with application to genetic fine mapping. J R Stat Society: Ser B. (2020) 82:1273–300. doi: 10.1111/rssb.12388. PMID: 37220626 PMC10201948

[24] Benner C Spencer CCA Havulinna AS Salomaa V Ripatti S Pirinen M . FINEMAP: efficient variable selection using summary data from genome-wide association studies. Bioinformatics. (2016) 32:1493–501. doi: 10.1093/bioinformatics/btw018. PMID: 26773131 PMC4866522

[25] Weissbrod O Hormozdiari F Benner C . Functionally informed fine-mapping and polygenic localization of complex trait heritability. Nat Genet. (2020) 52:1355–63. doi: 10.1038/s41588-020-00735-5. PMID: 33199916 PMC7710571

[26] Berisa T Pickrell JK . Approximately independent linkage disequilibrium blocks in human populations. Bioinformatics. (2016) 32:283–5. doi: 10.1093/bioinformatics/btv546. PMID: 26395773 PMC4731402

[27] 1000 Genomes Project Consortium . A global reference for human genetic variation. Nature. (2015) 526:68–74. doi: 10.1038/nature15393. PMID: 26432245 PMC4750478

[28] Miyanishi M Tada K Koike M Uchiyama Y Kitamura T Nagata S . Identification of Tim4 as a phosphatidylserine receptor. Nature. (2007) 450:435–9. doi: 10.1038/nature06307. PMID: 17960135

[29] Boada-Romero E Martinez J Heckmann BL Green DR . The clearance of dead cells by efferocytosis. Nat Rev Mol Cell Biol. (2020) 21:398–414. doi: 10.1038/s41580-020-0232-1. PMID: 32251387 PMC7392086

[30] Karczewski KJ Francioli LC Tiao G . The mutational constraint spectrum quantified from variation in 141,456 humans. Nature. (2020) 581:434–43. doi: 10.1038/s41586-020-2308-7. PMID: 32461654 PMC7334197

[31] Dorner BG Dorner MB Zhou X Opber C Seidl T Kroczek RA . Selective expression of the chemokine receptor XCR1 on cross-presenting dendritic cells determines cooperation with CD8+ T cells. Immunity. (2009) 31:823–33. doi: 10.1016/j.immuni.2009.08.027. PMID: 19913446

[32] Zhou R Zhang Q Xu P . TBK1, a central kinase in innate immune sensing of nucleic acids and beyond. Acta Biochim Biophys Sin. (2020) 52:757–67. doi: 10.1093/abbs/gmaa051. PMID: 32458982

[33] Hayden MS Ghosh S . Shared principles in NF-kappaB signaling. Cell. (2008) 132:344–62. doi: 10.1016/j.cell.2008.01.020. PMID: 18267068

[34] Avsec Z Agarwal V Visentin D Ledsam JR Grabska-Barwinska A Taylor KR . Effective gene expression prediction from sequence by integrating long-range interactions. Nat Methods. (2021) 18:1196–203. doi: 10.1038/s41592-021-01252-x. PMID: 34608324 PMC8490152

[35] Huang C Shuai RW Baokar P . Personal transcriptome variation is poorly explained by current genomic deep learning models. Nat Genet. (2023) 55:2056–9. doi: 10.1038/s41588-023-01574-w. PMID: 38036790 PMC10703684

