# Supplementary figures and images for: Multi-layered functional genomics prioritizes candidate effectors and regulatory mechanisms of ankylosing spondylitis
Source: Front Immunol. 2026 Jun 1;17:1859310. doi: 10.3389/fimmu.2026.1859310 (PMC13265330; doi:10.3389/fimmu.2026.1859310)

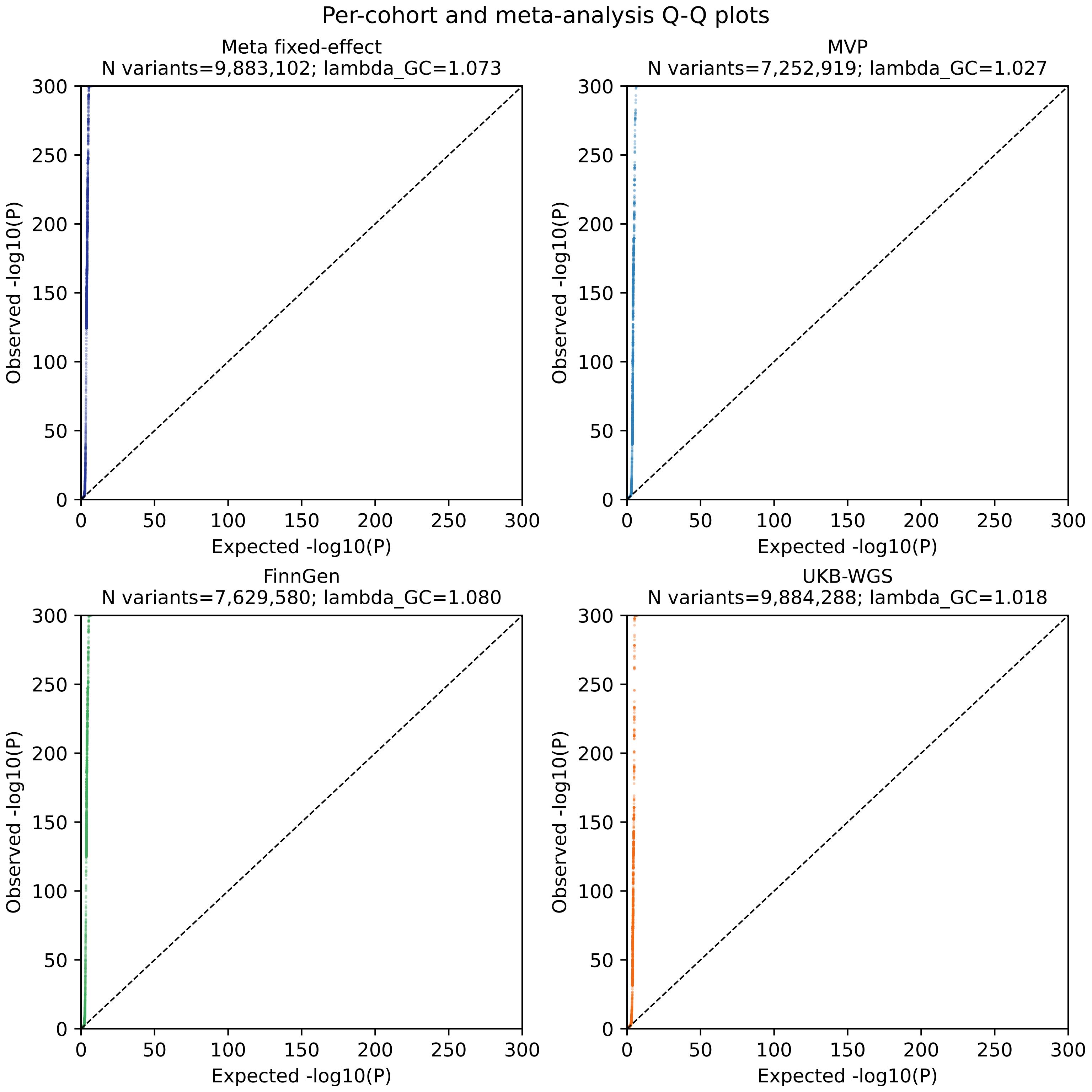

Supplement: Supplementary file 2 [file Image1.jpg]

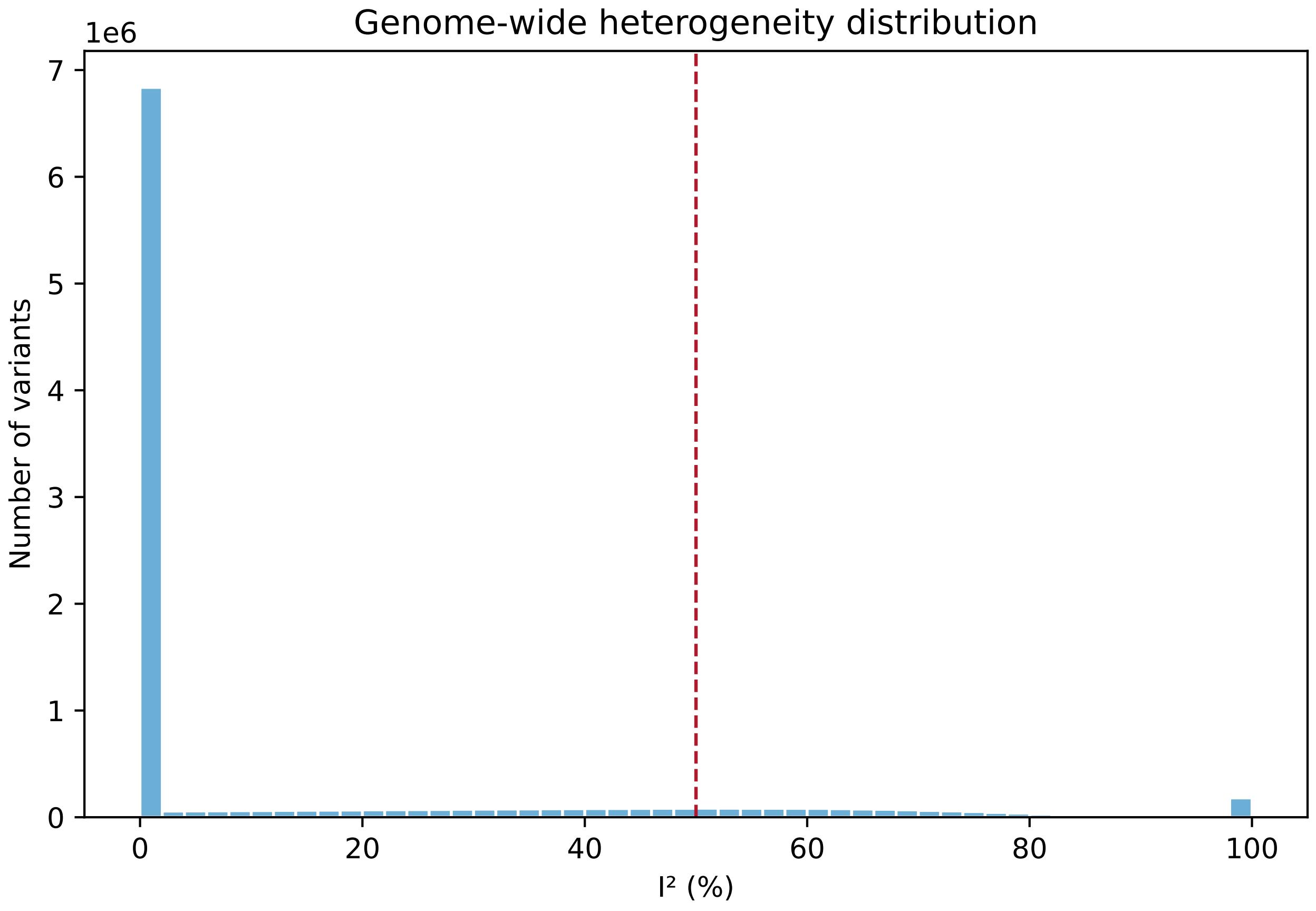

Supplement: Supplementary file 3 [file Image2.jpg]
